# The First Virtual Cranial Endocast of a Lungfish (Sarcopterygii: Dipnoi)

**DOI:** 10.1371/journal.pone.0113898

**Published:** 2014-11-26

**Authors:** Alice M. Clement, Per E. Ahlberg

**Affiliations:** Department of Organismal Biology, Evolutionary Biology Centre, Uppsala University, Uppsala, Sweden; Institute of Botany, China

## Abstract

Lungfish, or dipnoans, have a history spanning over 400 million years and are the closest living sister taxon to the tetrapods. Most Devonian lungfish had heavily ossified endoskeletons, whereas most Mesozoic and Cenozoic lungfish had largely cartilaginous endoskeletons and are usually known only from isolated tooth plates or disarticulated bone fragments. There is thus a substantial temporal and evolutionary gap in our understanding of lungfish endoskeletal morphology, between the diverse and highly variable Devonian forms on the one hand and the three extant genera on the other. Here we present a virtual cranial endocast of *Rhinodipterus kimberleyensis*, from the Late Devonian Gogo Formation of Australia, one of the most derived fossil dipnoans with a well-ossified braincase. This endocast, generated from a Computed Microtomography (µCT) scan of the skull, is the first virtual endocast of any lungfish published, and only the third fossil dipnoan endocast to be illustrated in its entirety. Key features include long olfactory canals, a telencephalic cavity with a moderate degree of ventral expansion, large suparaotic cavities, and moderately enlarged utricular recesses. It has numerous similarities to the endocasts of *Chirodipterus wildungensis* and *Griphognathus whitei*, and to a lesser degree to *'Chirodipterus' australis* and *Dipnorhynchus sussmilchi*. Among extant lungfish, it consistently resembles *Neoceratodus* more closely than *Lepidosiren* and *Protopterus*. Several trends in the evolution of the brains and labyrinth regions in dipnoans, such as the expansions of the utricular recess and telencephalic regions over time, are identified and discussed.

## Introduction

Dipnoans, or lungfish, are a clade of lobe-finned fish whose stem members can be found as far back as the earliest Devonian, more than 400 million years ago [1] [2]. Today the crown group contains only three extant genera (*Protopterus, Lepidosiren, Neoceratodus*), but like many other sarcopterygians, lungfish were most diverse early in their history with over 90 species known from the Devonian alone [3]. They have a rich and informative fossil record, particularly for the Devonian part of their history [4]. The phylogenetic position of lungfish has long been contentious, but they are now accepted on both molecular and morphological grounds as the living sister taxon to the tetrapods [5]–[9]. Their phylogenetic position makes lungfish particularly salient for evolutionary-developmental and comparative morphological studies with respect to tetrapods, as well as more broadly within an early vertebrate framework.

Of the extant dipnoans, the neoceratodontid lineage is thought to have diverged from the lepidosirenids (*Protopterus* and *Lepidosiren*) as long ago as the Permian [10], [11]. As would be expected, the lepidosirenid lungfish are more similar to each other than to *Neoceratodus*, the sole remaining species of the Neoceratodontidae. The two lungfish families differ strikingly in a number of morphological features, including their overall body shape, both body and paired fins, skull roof, dentition, and in a number of soft tissue elements including their nervous systems [12]–[18].

**Figure 1:**
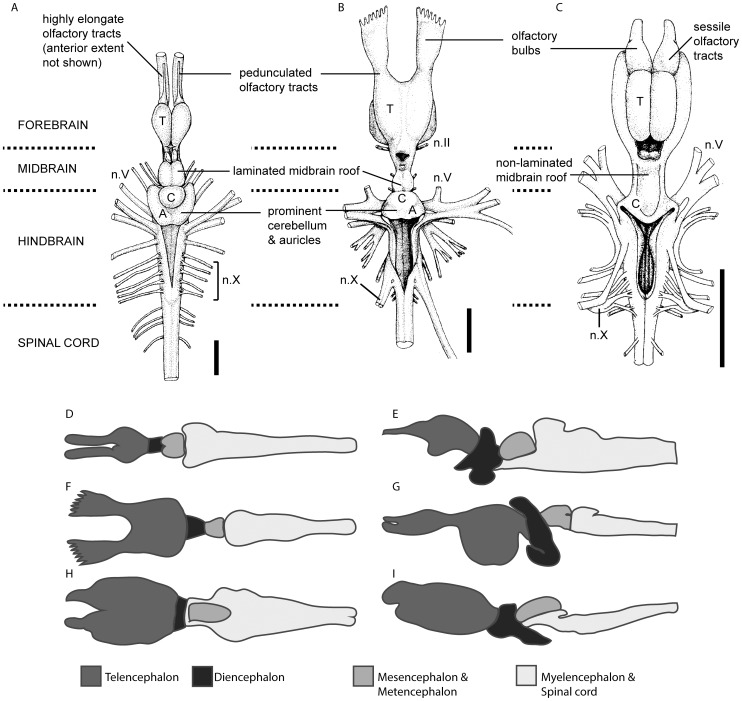
Gross morphology of extant sarcopterygian fish brains. A, *Latimeria*; B, *Neoceratodus*; and C, *Protopterus* in dorsal view, modified from Northcutt [16]; and schematic representations of extant sarcopterygian brains showing brain divisions, *Latimeria* in D, dorsal and E, lateral view (adapted from Niewenhuys [19]); *Neoceratodus* in F, dorsal (adapted from Northcutt [16], [17]) and G, lateral view (adapted from Holmgren and van der Horst [14]); and *Protopterus* in H, dorsal view (adapted from Northcutt [16]) and I, *Lepidosiren* in lateral view (adapted from Collin [18]). Scale bar equals 1 cm. Abbreviations: A, auricle; C, cerebellum; n.II, optic nerve; n.V, trigeminal nerve; n.X, vagus nerve; T, telencephalon.

The brains of lepidosirenid lungfish differ quite remarkably from that of *Neoceratodus*. In fact, more similarities exist between the brains of lepidosirenid lungfish and lissamphibians, whereas the adult *Neoceratodus* brain actually more closely resembles that of the coelacanth, *Latimeria* [16], [18]–[20]. *Latimeria* and *Neoceratodus* both possess an extensive cerebellum, distinct laterally paired auricles, and a large and laminated midbrain roof, in contrast to lepidosirenids and lissamphibians that instead have a small cerebellum, auricles that can only be distinguished from the corpus histologically, and a relatively small, non-laminated midbrain roof (Figure 1). Similarly, the olfactory bulbs are pedunculate (connected by olfactory tracts) in *Latimeria* and *Neoceratodus*, whereas they are sessile in lissamphibians and the lepidosirenid lungfish [16]. This character distribution is incongruent with the well-supported monophyly of Dipnoi, suggesting that lungfish brain evolution has involved a significant degree of homoplasy, either in the form of convergence between lepidosirenids and tetrapods or between *Neoceratodus* and *Latimeria*. Fossil endocranial data from the dipnoan stem group are needed to polarize these evolutionary transformations.

The first illustrated endocast of any Devonian lungfish was that of *Chirodipterus wildungensis* [21]. Later, a number of uncrushed specimens were used to reconstruct the neurocranial cavity of *Dipnorhynchus* illustrating the relationship between the braincase and the canals for nerves and blood vessels [22], [23], and again similarly for *Holodipterus* from a single specimen [24]. Partial endocasts of the extinct dipnomorphs *Youngolepis* [25] and *Powichthys* [26], and the porolepiform *Glyptolepis* [27] are also known, as are those of primitive tetrapodomorphs such as *Tungsenia* [28]and *Eusthenopteron* [29].

Here we present an investigation by x-ray computed tomography (µCT) of the braincase and cranial cavity of *Rhinodipterus kimberleyensis* [30], [31], a recently described stem-group dipnoan from the Late Devonian Gogo Formation in northern Western Australia. The Gogo Formation is a lagerstätte known for its perfect 3D preservation and high diversity of taxa [32], [33]. *Rhinodipterus* is one of the most crownward stem dipnoans with a near-complete ossified braincase known. Lungfish endoskeletons have undergone a radical reduction in ossification since the Devonian, possibly as a result of paedomorphosis [34], with the result that many post-Devonian lungfish are known only from dermal bones and teeth, and none preserves a complete three-dimensional braincase.

The reconstruction of internal structures in vertebrate fossils has undergone a technical revolution in recent years with the shift from physical serial sectioning or grinding [35], [36] to tomographic techniques based on X-rays or other types of radiation that penetrate the specimen [37]–[42]. While brains themselves are only very rarely preserved [43], the study of cranial endocasts as a proxy for brain morphology [44] is a thriving field. Of course, one must take care when interpreting endocasts, especially those of fishes where the brain can incompletely fill the neurocranial cavity [14], [43]. Nevertheless, endocasts of exquisitely preserved fossils can provide much information about the relative sizes of different regions of the brain, and thus provide a basis for tentative inferences about the sensory and motor capabilities of the animal [45]. Endocasts are also highly character rich, highlighting an underutilized source of anatomical traits for comparative morphology and cladistic analysis. The cranial endocast of *Rhinodipterus* presented here is the first near-complete cranial cavity of a stem lungfish to be imaged tomographically. It demonstrates both the utility of the technique and the high quality of the anatomical data obtainable from Gogo Formation material.

## Materials and Methods

### *Rhinodipterus kimberleyensis*

Clement 2012 (WAM 09.6.149) from the Frasnian Gogo Formation, was acid-prepared by Prof. John A. Long (MV), and is currently housed at the West Australian Museum, Perth. The uncrushed specimen was scanned at the Australian National University (ANU) High Resolution X-ray Computed Tomography facility [46], with a scan resolution of 55.5 microns. Three-dimensional modeling and segmentation was completed using the software *VGStudio Max*, version 2.2 (Volume Graphics Inc., Germany). Permits were not required for the described study, which complied with all relevant regulations.

We use the following brain terminology herein: telencephalon and diencephalon comprise the forebrain, the mesencephalon the midbrain, and the hindbrain is composed of the metencephalon and myelencephalon. Anteriorly, housed within the olfactory canal, the olfactory tract is an extension of the brain itself (not a nerve). The tract can be expanded into an olfactory bulb anteriorly, usually contained within a nasal sac or cavity [47].

## Description

**Figure 2:**
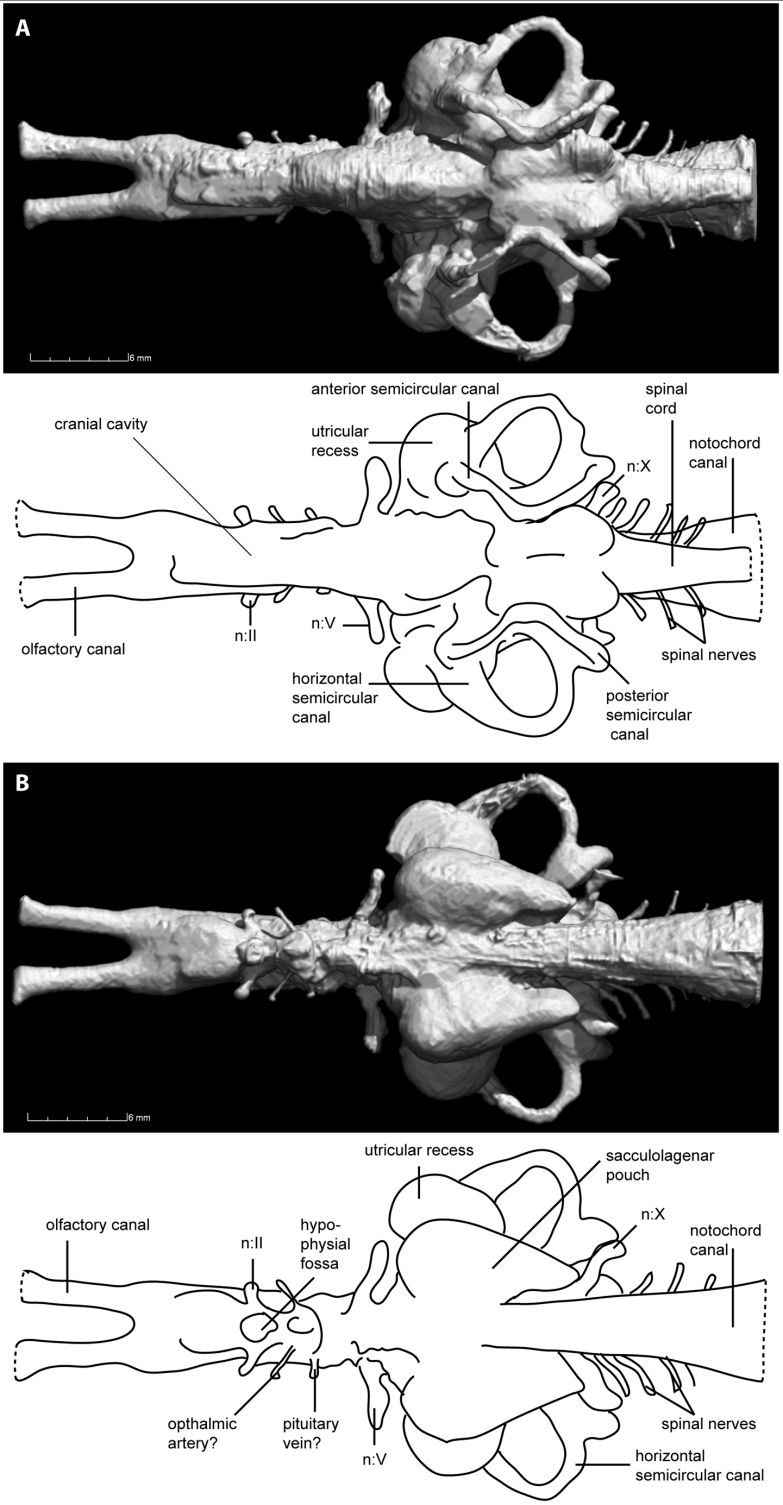
Virtual reconstruction and schematic illustrations of *Rhinodipteus kimberleyensis* (WAM 09.6.149) cranial endocast in A, dorsal; and B, ventral views. Abbreviations: n.II, optic nerve; n.V, trigeminal nerve; n.X, vagus nerve.

**Figure 3:**
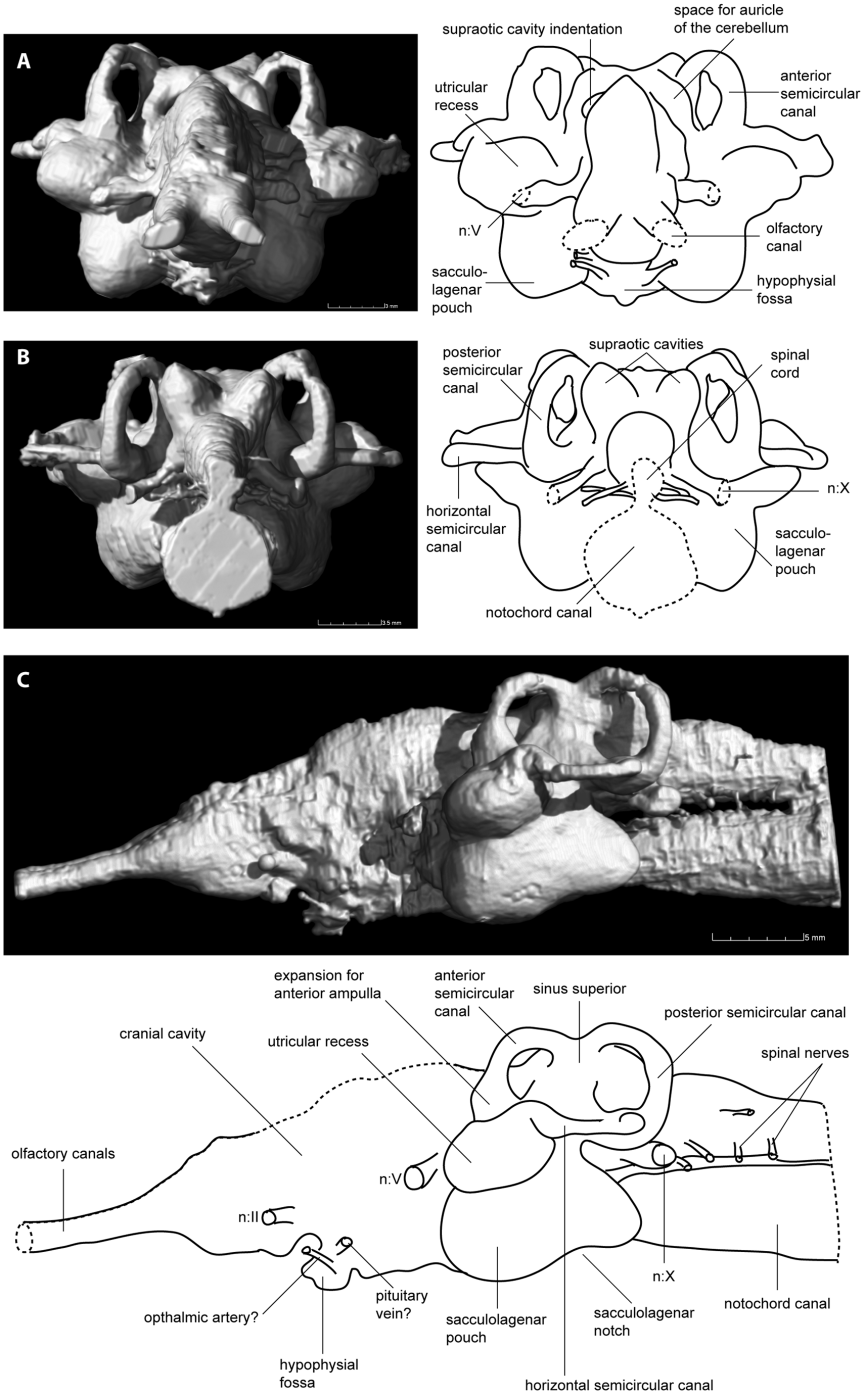
Virtual reconstruction and schematic illustrations of *Rhinodipteus kimberleyensis* (WAM 09.6.149) cranial endocast in A, anterior; B, posterior; and C, lateral views. Abbreviations: n.II, optic nerve; n.V, trigeminal nerve; n.X, vagus nerve.

Figures 2 and 3 show a virtual endocast of the neurocranial cavity of *Rhinodipterus kimberleyensis*. It measures approximately 45 mm in length, and 20 mm at the widest point across the horizontal semicircular canals. The neurocranium is incomplete anteriorly (snout tip missing), and damaged dorsally in the orbito-temporal region [31], but otherwise it is complete and uncompressed with many smaller canals for cranial nerves or blood vessels identifiable. The posterior portion of the neural cavity corresponding to the myelencephalic region or hindbrain (including the labyrinth region) is particularly well preserved.

The telencephalic region is long (at least longer than 14 mm) and slightly broader than the midbrain cavity (by approx. 0.5 mm). Anteriorly, the endocast has long, slender olfactory canals that are circular in cross-section. They lie close to each other and diverge only narrowly (Fig. 2). The anterior portion of the snout is missing, so the nasal capsules are unknown. The olfactory canals merge with the telencephalic cavity proper in the posterior third of the telencephalic region; relative to the canals, the cavity bulges ventrally as well as (to a lesser degree) laterally and dorsally.

Posterior to the telencephalon, the anterior margin of the diencephalic region is marked by the canals for the optic nerves (n.II) that emerge into the orbits. A small, smoothly rounded ventral protuberance, separated from the telencephalic cavity by a distinct notch, represents the hypophysial fossa (Fig. 2B, 3A,C). Two pairs of canals for blood vessels emerge from the hypophysial fossa; anteriorly possibly for the ophthalmic artery and posteriorly possibly for the pituitary vein (Fig. 2B). Unfortunately this region of the braincase has suffered some damage dorsally, so canals for nerves III and IV that should emerge from the dorsal part of the cranial cavity are not preserved. The diencephalic, mesencephalic and metencephalic regions are strongly laterally compressed (between 3.5–5.1 mm in width), all slightly narrower than the telencephalic region (≈5.5 mm).

Wide canals for the trigeminal nerves (n.V) anterior to the labyrinth region mark the anterior boundary of the myelencephalic region (Fig. 2). While the mesencephalic and metencephalic regions are short, the myelencephalon is longer (almost the same length as all three preceding regions combined, ≈10 mm). Dorsally, the supraotic cavities are well defined and carry broad, bulging anterior and posterior prominences (Figs. 2A, 3B). In anterior view two deep indentations where the supraotic cavities attach to the cranial cavity can be seen (Fig. 3A).

The semicircular canals have already been described in *R. kimberleyensis* [31], the sacculolagena and utricular recess could not be modeled at that time and were thus neglected in that description. These regions are in fact ossified, well preserved and visible from the scan data, measuring close to 20 mm across their widest point (Fig. 2). The sinus superior is laterally compressed and stands vertically (Fig. 3C). Both the utricular recess and sacculolagena are large, rounded structures. The sacculolagena of *Rhinodipterus* protrudes ventrally below the notochordal chamber, and there is a distinct ventral notch in the sacculolagena (Fig. 3C). An expansion in the anterior semicircular canal exists for the anterior ampulla, but the absence of a significant expansion for the posterior ampulla on the posterior semicircular canal [31] is confirmed.

On either side of the specimen, behind the labyrinth region, a thick canal for the vagus nerve (n.X) extends posterolaterally. Posterior to the vagus nerve, five narrow canals for spinal nerves can be seen extending laterally from the cranial cavity (Figs. 2, 3B,C). Moving from the posterior margin of the labyrinth region to the posterior end of the endocast, the cranial cavity gradually narrows by nearly half its width, and also becomes lower. At its most posterior point the cranial cavity has a narrow upright-oval cross-section (Fig. 3B).

The canal for the notochord is wide and circular in cross-section posteriorly. It gradually narrows and becomes shallower towards the anterior, while simultaneously flexing ventrally to accommodate the ventral expansion of the mesencephalic-metencephalic region of the cranial cavity; its anterior end lies just posterior to the hypophyseal fossa (Fig. 2B).

## Discussion

### Comparisons with other taxa

Obviously care must be taken when interpreting cranial cavity endocasts, especially as the shape of fish brains are generally not constrained as tightly as in other groups such as mammals and birds [48], [49]. However, Stensiö [50] noted that the telencephalic and diencephalic regions in *Neoceratodus* very closely reflected the shape of the cranial cavity. Moreover, no part of the brain can be larger than the cavity that houses it, and this simple fact places strong constraints on the inferred brain morphology.

When the cranial cavity of *Rhinodipterus* is compared with the brain morphology of the extant lungfish (Figure 1), substantial differences in telencephalic morphology immediately become apparent. All the extant genera have large telencephalic lobes: in *Protopterus* and *Lepidosiren* they are oblong [12], [16], [18], whereas in *Neoceratodus* they are rounder, and very deep ventrally [14]. The olfactory bulbs are sessile in the lepidosirenids but separated from the telencephalon by short olfactory tracts in *Neoceratodus*; in all three genera the olfactory bulbs (or tracts) attach anterodorsally on the telencephalon. In *Rhinodipterus* the telencephalic lobes must have been much smaller, though the predominantly ventral expansion of the telencephalic cavity suggests a shape somewhat like the brain in *Neoceratodus*, and the olfactory tracts must have been long.

The middle part of the cranial cavity of *Rhinodipterus* is less informative, partly because it is damaged dorsally, but it nevertheless allows some comparisons to be made with the extant genera. The hypophysis appears to have been oriented ventrally as in *Neoceratodus*, not posteroventrally as in *Lepidosiren* [14], [18] and *Protopterus* [12]. The shape of the mesencephalic-metencephalic cavity suggests the presence of quite large auricles extending forward on either side of a raised middle region of the brain, as in *Neoceratodus* or *Latimeria*. The raised middle region would have comprised the optic tectum and cerebellum, but unfortunately nothing can be said about their morphology and relative proportions beyond the fact that they were narrow in dorsal view.

**Figure 4:**
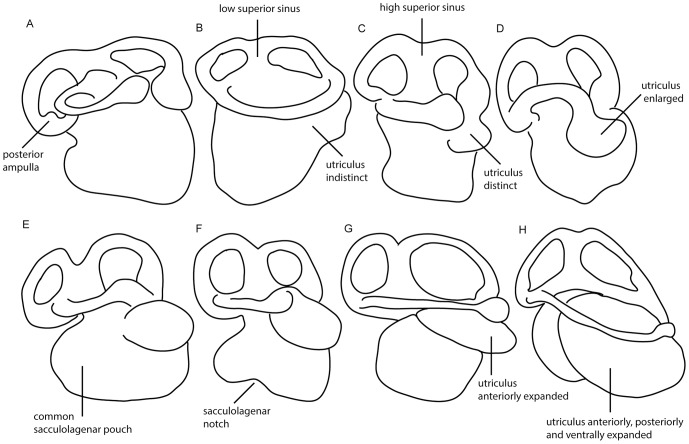
Labyrinth region morphology of eight dipnomorphs. A, *Youngolepis praecursor* (from Chang [25], fig. 19); B, *Dipnorhynchus sussmilchi* (from Campbell and Barwick [22], fig. 25); C, ‘*Chirodipterus’ australis* (from Miles [52], fig. 48); D, *Chirodipterus wildungensis* (from Säve-Söderbergh [21], fig. 9); E, *Griphognathus whitei* (from Miles [52], fig. 46); F, *Rhinodipterus kimberleyensis* (WAM 09.6.149); G, *Neoceratodus forsteri*; and H, *Protopterus annectens* (both from Retzius [51], plate XXIV). Not to scale, anterior is to the right.

The labyrinth region of modern lungfish are distinguished from those of other gnathostomes by possession of an enlarged utriculus [51], [52]. In the lepidosirenids this is enormous, as big as or bigger than the (undifferentiated) sacculolagena, whereas *Neoceratodus* shows a less extreme morphology with a somewhat smaller utriculus (Figure 4). *Rhinodipterus* has a labyrinth cavity resembling that of *Neoceratodus*, except that its utricular cavity is slightly smaller and does not extend so far posteriorly (Figure 4F,G). The sinus superior is tall and narrow, as in all modern lungfish. The only surprising feature of the ear of *Rhinodipterus* is the shallow notch, situated approximately between the saccular and lagenar portions, which is not matched either by modern lungfish or by other fossil stem lungfish (see below).

As can be seen, these comparisons yield a remarkably consistent picture: on every point, the endocast of *Rhinodipterus* is more similar to the brain of *Neoceratodus* than to the lepidosirenids. Given that *Rhinodipterus* is a stem lungfish, this strongly suggests that the similarities reflect retained primitive characters in *Neoceratodus*, relative to a more derived morphology in lepidosirenids. Comparison with other members of the lungfish stem group, and with the non-dipnoan sarcopterygians *Eusthenopteron*, *Tungsenia* and *Spodichthys*, support this conclusion and add further information about character evolution in the early part of the lungfish stem lineage.

In *Griphognathus whitei* from the Gogo Formation, an exact contemporary of *Rhinodipterus* with a similar long-snouted morphology, parts of the cranial cavity and labyrinth spaces (Fig. 4E) have been described from acid-prepared specimens [52]. The information is more limited than that for *Rhinodipterus*, but certain comparisons can be made. The olfactory canals, which are very long, merge posteriorly with a short and narrow telencephalic cavity ([52] figs. 56, 61, 63). Proportionately, this cavity appears somewhat smaller than that of *Rhinodipterus*, with a less pronounced ventral bulge ([52] fig. 10). There is a large anterodorsally directed pineal recess ([52] fig. 10); this region of the cranial cavity is unfortunately damaged in *Rhinodipterus*, so the two taxa cannot be compared in this respect. The labyrinth cavity and supraotic cavity of *Griphognathus* have been figured in some detail ([52] fig. 46). The supraotic cavity is broadly similar to that of *Rhinodipterus* but is not as bulbous and sac-like. However, the labyrinth cavity has an enlarged utricular recess (Fig. 4E), very similar to that of *Rhinodipterus*.

*Holodipterus gogoensis*, also from the Gogo Formation, shows the shape of the braincase particularly well in one weathered specimen (ANU 49102) [24]. The authors have identified numerous nerve canals and approximate endocranial proportions. *Holodipterus* shows two short, robust, and broadly diverging olfactory canals ([24] fig. 6b), contrasting with the narrower and more elongate canals in *Rhinodipterus*. The space for the telencephalon is relatively short and does not allow for any considerable ventral expansion. Like *Griphognathus, Holodipterus* also exhibits a large, anterodorsally directed pineal recess ([24] fig. 6b). The labyrinth region more closely resembles *Chirodipterus wildungensis* (Fig. 4C) with space for a high superior sinus but lacking a distinctively enlarged utriculus, such as that in *Rhinodipterus*.

**Figure 5:**
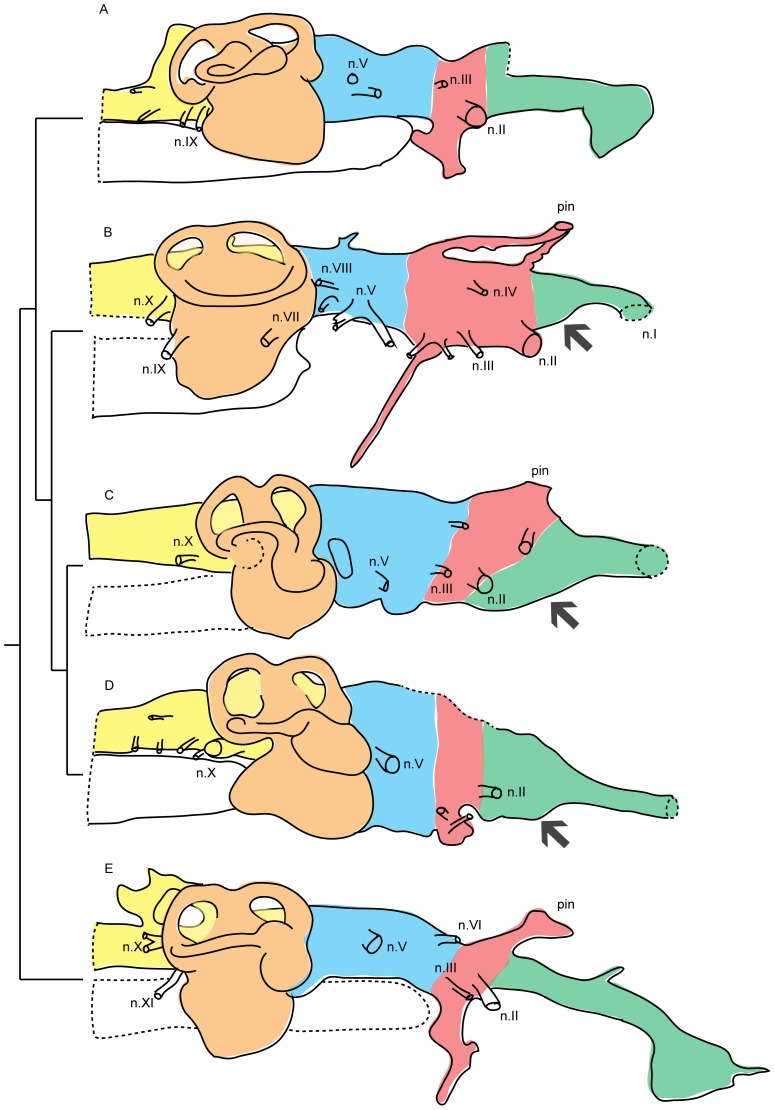
A, *Youngolepis praecursor* (from Chang [25], fig. 19); B, *Dipnorhynchus sussmilchi* (from Campbell and Barwick [22], fig. 25); C, *Chirodipterus wildungensis* (from Säve-Söderbergh [21], fig. 9); D, *Rhinodipterus kimberleyensis* (WAM 09.6.149); and E, *Eusthenopteron foordi* (from Jarvik [69], figs. 57, 59). Not to scale, anterior is to the right, grey arrows indicate telencephalic ventral expansion. Abbreviations: n.I, olfactory nerve; n.II, optic nerve; n.III, oculomotor nerve; n.IV, trochlear nerve; n.V, trigeminal nerve; n.VII, facial nerve; n.VIII, auditory nerve; n.IX, glossopharyngeal nerve; n.X, vagus nerve; pin, pineal gland. Colour key: green, telencephalic region; red, diencephalic region; blue, mesencephalic region; yellow, metencephalic and myelencephalic regions; and orange, labyrinth region.

In *Chirodipterus wildungensis* (Figs. 4C, 5C), which comes from the latest Frasnian [53] of Bad Wildungen, Germany, and is thus the youngest of the fossil lungfish discussed here, the telencephalic cavity appears to be somewhat larger and deeper than that of *Rhinodipterus*. The olfactory bulbs were pedunculate, though it is difficult to determine the exact length of the tracts. Dorsally there is a prominent pineal recess. The labyrinth cavity of *Chirodipterus* is similar to that of *Rhinodipterus* except that the utricular cavity is slightly smaller (Figures 4 and 5). Supraotic cavities have not been described.

*Chirodipterus australis* from the Gogo Formation, which has been figured in the same manner as *Griphognathus whitei* [52] shows some interesting differences from *Chirodipterus wildungensis*. The overall shape of the cranial cavity is similar, again with a well-developed pineal recess, but the telencephalic cavity appears to be proportionately shorter and the olfactory canals are long ([52] figs. 17, 66). In the labyrinth cavity, the utricular recess is much smaller than in the aforementioned taxa (Figure 4C,D) and the supraotic cavity is small and simple in shape ([52] fig. 47). As previously recognized [54], these and other features suggest that *‘Chirodipterus’ australis* is not closely related to *C. wildungensis* and should not be assigned to that genus, *‘C.’ australis* is probably a less crownward member of the stem group than *C. wildungensis*.

Campbell and Barwick [22] presented a reconstruction of the cranial endocast of *Dipnorhynchus sussmilchi*, a considerably earlier (Emsian, late Early Devonian) and less crownward member of the lungfish stem group than any of the aforementioned forms [54]. The reconstruction (Fig. 5B), which is based on observations of acid-prepared specimens, includes some surprising features such as an extremely deep hypophysial recess and widely separated origins for the pineal and parapineal organs. Nevertheless, we judge it to be reliable in most respects. The telencephalic region as a whole is remarkably short, especially in comparison with the long metencephalic-mesencephalic-diencephalic region. However, its proportions are broadly similar to those of *Rhinodipterus*, except that the olfactory canals are shorter and more divergent. As in *Rhinodipterus*, the olfactory canals emerge anterodorsally from the telencephalic cavity, which has a small but distinct ventral bulge (Fig. 5B). In the labyrinth cavity the utricular recess is not enlarged at all and can scarcely be distinguished as a separate entity (but note that a small differentiated utriculus is reconstructed in *Dipnorhynchus kurikae*, [23] figs. 4b,5c). The sinus superior is quite low and the supraotic cavity is much smaller than in *Rhinodipterus*.

The stem-group members discussed above are all forms that from a traditional ‘key character’ perspective would be (and have been) deemed conventional ‘fossil lungfish’: they possess major components of the modern lungfish character complex such as autostyly, absence of an intracranial joint, and a palatal bite. This contrasts with *Youngolepis*, *Powichthys* and porolepiforms -considered in the next section- which are assigned to the most basal part of the dipnoan stem group in the majority of recent analyses [28], [55] but are not so obviously lungfish-like. The ‘fossil lungfish’ are all undisputed members of the stem group [22], [55], [56] and are always recovered crownward to *Youngolepis*, *Powichthys* and porolepiforms.

The cranial cavity of *Youngolepis* (Fig. 5A) has been described in its entirety [25], whereas in *Powichthys* and porolepiforms (represented here by *Glyptolepis groenlandica*) the part posterior to the diencephalon is unknown [26], [27]. The telencephalic regions of all three taxa resemble each other, and differ from those of the ‘fossil lungfish’ as well as extant lungfish, on several points. The telencephalic cavity proper is very short, and the olfactory canals correspondingly long. Furthermore, there is no ventral expansion of the telencephalic cavity; insofar as there is a vertical expansion at all (in *Powichthys* and *Glyptolepis*, but not in *Youngolepis*) it is dorsal to the exits of the olfactory canals. In *Eusthenopteron* (Figure 5E), *Tungsenia* and *Spodichthys*, which are members of the tetrapod stem group and can thus be used as an outgroup in this context, the telencephalic cavity is longer than in *Youngolepis*, *Powichthys* and *Glyptolepis*, but there is again no obvious ventral expansion [28], [29], [57]. All six genera have large anterodorsally directed pineal recesses. The otic region of *Youngolepis* closely resembles that of *Eusthenopteron* (Fig. 5A,E): the utricular recess is not enlarged, the sinus superior is low, and the supraotic cavity resembles that of *Griphognathus*.

### Evolutionary implications

A number of interesting patterns of character distribution and polarity within the lungfish total group emerge when these morphologies are considered in a phylogenetic framework:

1. Primitively, the nasal cavities are separated from the telencephalic cavity by long olfactory canals. Although the exact location of the olfactory bulb within the cranial cavities of the fossil forms is not strongly constrained (see Jarvik 1980: fig. 89, for one attempt at reconstruction [29]), it is clear that pedunculate olfactory bulbs are primitive for the lungfish total group. Sessile bulbs are a derived feature of lepidosirenids.
2. The telencephalon proper is primitively short. All extant lungfish have a longer telencephalon than the stem group members. Lepidosirenids are more derived than *Neoceratodus* in this regard.
3. A ventral expansion of the telencephalon is primitively absent (as shown by *Youngolepis*, *Powichthys* and *Glyptolepis*), but begins to develop in conjunction with other aspects of lungfish morphology such as autostyly and a palatal bite. *Dipnorhynchus* and all more crownward stem lungfish have such an expansion, but it is smaller than that of modern lungfish.
4. The pineal and parapineal organs primitively reached close to the skull roof, though there may not have been a pineal foramen (a foramen is present in *Dipnorhynchus* and *Powichthys*, but absent in all other members of the lungfish total group). This condition is characteristic of all known stem group members (though unknown in *Rhinodipterus* because of specimen damage); the reduced size and height of the pineal-parapineal complex in extant lungfish is derived (as recognised by Stensiö 1963 [50]). Lepidosirenids are more derived than *Neoceratodus* in this regard.
5. An expanded utriculus develops gradually within the lungfish stem group, crownward of *Dipnorhynchus*. The utriculi of extant lungfish are larger than those of any stem group members, but once again the lepidosirenids are much more derived than *Neoceratodus*.

Together, these patterns build up a picture of rather modest, directional change during the early evolution of lungfish, centered principally on ventral telencephalic expansion and enlargement of the utriculus. This was a prolonged process, as shown by the substantial differences between even the most derived Devonian stem group members such as *Rhinodipterus*, and the extant lungfish. Stensiö's (1963: p. 82 [50]) assertion, based on *Chirodipterus wildungensis*, that the extant ‘dipnoan brain had evolved prior to the beginning of the Devonian and that since that time it has remained practically unchanged’ is incorrect.

It has long been recognized that the brains of the lepidosirenid lungfish more closely resemble those of lissamphibians, whereas the brains of *Neoceratodus* and *Latimeria* hold more similarities to each other [16], [18]–[20]. The data from *Rhinodipterus* and other stem lungfish demonstrate that the lissamphibian-like characteristics of lepidosirenids are convergent, at least where gross morphology is concerned. Indeed, there is a remarkable consistency about the characteristics of *Neoceratodus* and the lepidosirenids relative to *Rhinodipterus* and the other members of the stem group: *Neoceratodus* is always primitive relative to the lepidosirenids and displays no obvious autapomorphies at all. It is very difficult to determine the cause(s) of this apparent stasis; we simply draw attention to it here as a phenomenon worthy of investigation alongside the more widely known morphological conservatism of the coelacanth *Latimeria*. It is also interesting to note that the derived characteristics of the lepidosirenids largely represent further developments of trends that are already evident in the Devonian members of the lungfish stem group, most strikingly the expansion of the telencephalon and utriculus. The lepidosirenids, it seems, continued to follow the evolutionary directions that had been established early in the history of the lungfish, whereas the lineage leading to *Neoceratodus* entered a state of near-stasis shortly after it split from the lepidosirenid lineage during the Permian, about 277 million years ago [11].

### Functional significance

The neuroanatomy of extant lungfish allow us to draw some tentative conclusions about the functional significance of the telencephalic expansion. In *Neoceratodus* the part of the telencephalon that lies ventral to the plane of the olfactory tract consists entirely of the subpallium [14]. This region of the brain appears to deal substantially with olfaction, and we can probably conclude that the ventral expansion of the telencephalon during lungfish evolution related functionally to the development of an enhanced sense of smell [19]. Conversely, the mesencephalon, which receives visual input, is proportionately small in both stem and crown lungfish, relative to most actinopterygians and chondrichthyans.

There are surprisingly few studies on lobe-finned fish labyrinths, despite the likely insights they could provide into the transition of the early tetrapods [58]–[60]. Retzius [51] was the first to describe them in lungfish, Millot and Anthony [61] for the coelacanth. Later authors have expanded this work [62]–[65]. *Latimeria* is considered to have an labyrinth region “with tetrapod affinities” [62], whereas the dipnoan labyrinth has been described as having a mix of chondrichthyan and lissamphibian features [64].

The evolution of labyrinth morphology over time begs an obvious question: how does it relate to function and behaviour? The vestibular system in fish is involved in detecting body orientation in three dimensions, and respond to other cues such as gravity and acceleration. Clement [31] previously discussed semicircular morphology and its bearing on locomotive behaviour. The similarity between the labyrinth region of *Neoceratodus* and *Rhinodipterus* (Fig. 4F,G) drew her to conclude that some Late Devonian lungfish may have obtained similar functional abilities of the labyrinth as seen in extant taxa. Narrower semicircular canals increase sensitivity and are associated with more rapid swimming or accurate maneuverability [66]. The loss of ampullae, narrowing and heightening (effectively lengthening) of the canals -a trend seen over time in lungfish- almost certainly enhances sensitivity.

The functional significance of the striking utricular expansion in the Dipnoi is surprisingly difficult to determine. Early literature considered the utricule most important for postural control (particularly horizontal movement), whereas the sacculolagena was considered to have a more auditory role [67]. However, more recent literature suggests that all otolith organs may have both auditory and vestibular roles [67], often varying between different fish species [68]. Although conclusions can't be drawn without experimental evidence, the changing proportions of labyrinth organs likely indicate an altered sensitivity of the vestibular system with respect to posture control, and may or may not have had an additional role in hearing.

The form of the sacculolagenar pouch in *Rhinodipterus* is also worth mentioning. Most extant fish and amphibians possess separate saccular and lagenar maculae, however modern lungfish are unusual in this respect in having just one [65]. Primitive osteichthyans, including the earliest lungfish and tetrapods, also have a common sacculolagenar chamber as opposed to the large, separate lagena outgrowth seen in modern tetrapods [58] and actinopterygians [47]. Although *Rhinodipterus* maintains a common sacculolagenar pouch, its ventral margin shows a distinct ventral notch (Figs. 4F, 5D). This is particularly unusual as none of the other stem lungfish with labyrinth morphology known appear to do so, nor do the extant taxa.

## Conclusions

Our work represents the first virtual endocast of any lungfish. Despite being Late Devonian in age, *Rhinodipterus kimberleyensis* is one of the most derived near-complete fossil lungfish braincases known. Not surprisingly, of all the known stem lungfish endocasts, *Rhinodipterus* resembles the similarly-aged *Chirodipterus wildungesis* and *Griphognathus whitei* most closely [21], [52]. With respect to extant taxa, the endocast of *Rhinodipterus* consistently resembles the brain of *Neoceratodus* more than the lepidosirenids, suggesting *Neoceratodus* has retained more primitive characters than the other extant lungfish taxa. The stark difference in morphology between the Australian lungfish and the lepidosirenids is again noted. The early evolution of the lungfish stem group is characterized by expansion of the ventral part of the telencephalon, possibly related to evolution of an enhanced sense of smell, and slightly later, expansion of the utricular recess. Undoubtedly these changes in labyrinth proportions relate to increased sensitivity for some sensory input, whether it be acceleration, gravitational or auditory, or a combination of all three.
